# *Cryptosporidium geckonae* n. sp. (Apicomplexa: Cryptosporidiidae) in geckos

**DOI:** 10.1186/s13071-025-07068-4

**Published:** 2025-10-21

**Authors:** Veronika Zikmundová, Nikola Holubová, Jana Fenclová, Bohumil Sak, Michael Rost, Agnieszka Gomułkiewicz, Roman Konečný, John McEvoy, Lihua Xiao, Martin Kváč

**Affiliations:** 1 Institute of Parasitology, Biology Centre of the Academy of Sciences of the Czech Republic, v.v.i., Branišovská 31, 370 05 České Budějovice, Czech Republic; 2 Faculty of Agriculture and Technology, University of South Bohemia in České Budějovice, České Budějovice, Czech Republic; 3 Division of Histology and Embryology, Department of Human Morphology and Embryology, Wroclaw Medical University, Wroclaw, Poland; 4 Department of Microbiological Sciences, North Dakota State University, Fargo, ND USA; 5 State Key Laboratory for Animal Disease Control and Prevention, Center for Emerging and Zoonotic Diseases, College of Veterinary Medicine, South China Agricultural University, Guangzhou, China

**Keywords:** Biology, Course of infection, Cryptosporidiosis, Oocyst size, Phylogeny, Genetic diversity, Gecko

## Abstract

**Background:**

*Cryptosporidium* is a globally prevalent parasite that infects the gastrointestinal tract of a large number of vertebrates. The *Cryptosporidium* sp. lizard genotype is found almost exclusively in geckos but has also been detected in a corn snake. In this study, the biology and genetic variability of the lizard genotype were investigated.

**Methods:**

The genetic variability of the *Cryptosporidium* sp. lizard genotype was analysed by polymerase chain reaction (PCR) and Sanger sequencing of the *SSU* rRNA, actin, *HSP70* and *COWP* genes. Its biological characteristics, including oocyst size, tissue tropism and associated pathology, were investigated using light microscopy, electron microscopy and histological techniques. Experimental infections were performed to assess host specificity and infectivity.

**Results:**

Phylogenetic analysis of the target genes confirmed that the *Cryptosporidium* sp. lizard genotype is genetically distinct from other *Cryptosporidium* species. Experimental infections showed that chickens, mice and corn snakes were not susceptible to this genotype, as no infection was detected. In contrast, leopard geckos began to excrete oocysts between 6 and 8 days post-infection, although they showed no clinical symptoms. Excretion of oocysts continued for over 200 days. Oocysts isolated from naturally infected leopard and smooth knob-tailed geckos were morphometrically identical to those from experimentally infected leopard geckos and measured 5.89 × 4.79 μm. The parasite mainly colonised the stomach, where the highest intensity of infection was observed, but was also found throughout the intestine and in the lungs.

**Conclusions:**

Due to the distinct genetic differences, host range and tissue tropism, we propose the name *Cryptosporidium geckonae* n. sp. for this organism, which was previously referred to as *Cryptosporidium* sp. lizard genotype.

**Graphical Abstract:**

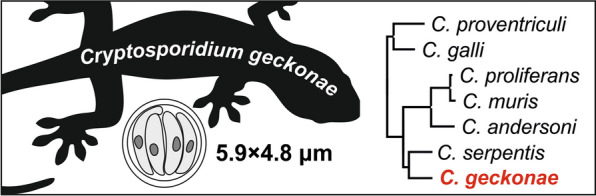

## Background

*Cryptosporidium* is a worldwide genus of unicellular parasites that cause diseases in mammals, birds, fish, reptiles and amphibians. Cryptosporidiosis, the disease caused by *Cryptosporidium*, can range from asymptomatic infections to severe, persistent watery diarrhoea without blood, often leading to dehydration and, in some cases, fatal outcomes [1]. The severity and course of the infection depend on the *Cryptosporidium* species and host factors such as the immune system and the age of the infected individual [2–4]. In the last 20 years, our knowledge regarding the genetic diversity and biology of these parasites has increased considerably. Currently, 47 valid species of the genus *Cryptosporidium* are known, and hundreds of so-called genotypes have been discovered on the basis of genetic divergence, mainly of the small subunit ribosomal RNA (*SSU*), from known species and other genotypes [5–8]. There are not sufficient genetic and biological data to recognise these genotypes as new species. Of the 48 described species, only *Cryptosporidium varanii* (junior syn. = *Cryptosporidium saurophilum*) and *Cryptosporidium serpentis* have been described in lizards. However, several genotypes have been described in these hosts, including the *Cryptosporidium* sp. lizard genotype [9–16].

The *Cryptosporidium* sp. lizard genotype was first described in the leopard gecko (*Eublepharis macularius*) in the Czech Republic as *Cryptosporidium* sp. 1655 [17]. It was later described under different names in the same host and in the corn snake (*Pantherophis guttatus*) in Austria, the common frog-eyed gecko (*Teratoscincus scincus*) in Japan and Kuroiwa’s ground gecko (*Goniurosaurus kuroiwae sengokui*) in Japan (Table 1). In the case of Kuroiwa’s ground gecko, this genotype was tentatively designated as *C. serpentis*, to which the *Cryptosporidium* sp. lizard genotype is phylogenetically closely related.

**Table 1:** Occurrence of *Cryptosporidium geckonae* n. sp. (previously known as *Cryptosporidium* sp. lizard genotype) in reptiles worldwide and in this study detected on the basis of molecular tools amplifying partial sequences of *Cryptosporidium* small subunit ribosomal RNA (*SSU*), actin, *Cryptosporidium* oocyst wall protein *(COWP*) and 70-kDa heat shock protein (*HSP70*) genes

Host	Country	Locus (GenBank ID)	GenBank name	No. positive/no. screened	Reference
Smooth knob-tailed gecko (*Nephrurus levis*)	Czech Republic	*SSU* (PV535941) actin (PV550915) *HSP70* (PV550920) *COWP* (PV550925)	*Cryptosporidium geckonae*	1/1	This study
Leopard gecko (*Eublepharis macularius*)	Czech Republic	*SSU* (PV535942) actin (PV550916) *HSP70* (PV550920) *COWP* (PV550926)	*Cryptosporidium geckonae*	4/4	This study
		*SSU* (AY120915) actin (AY120932)	*Cryptosporidium* sp. 1665	1/1	[17]
	Austria	*SSU* (HM750253)	*Cryptosporidium* sp. *Pantherophis*/659209	1/462	[11]
Corn snake (*Pantherophis guttatus*)	Austria	*SSU* (HM750254)	*Cryptosporidium sp. Eublepharis*/515708	1/1	[11]
Common frog eye gecko (*Teratoscincus scincus*)	Japan	*SSU* (LC010214) actin (LC010216)	*Cryptosporidium sp. BAH7*	1/1	[34]
Kuroiwa’s ground gecko (*Goniurosaurus kuroiwae sengokui*)	Japan	*SSU* (LC759647)	*Cryptosporidium serpentis* Tokashiki1	1/1	[35]

The aim of the present study was to molecularly and biologically characterise the *Cryptosporidium* sp. lizard genotype. The results of experimental infections, morphometric oocyst analyses, localisation of the infection in the host and comparative molecular analyses indicate that it is a distinct species. Therefore, we propose that this genotype be recognised as a new species of the genus *Cryptosporidium*.

## Methods

### Specimens and data collection

Two isolates of the *Cryptosporidium* sp. lizard genotype were obtained from naturally infected adult reptiles: a smooth knob-tailed gecko (*Nephrurus levis*; isolate liz_CZK_1.0) and a leopard gecko (*E. macularius*; isolate liz_CZK_2.0). The animals originated from different breeders and were the offspring of captive-bred parents. The presence of oocysts in their faecal samples was confirmed by aniline-carbol-methyl violet (ACMV) staining [18], and the genotype was determined by polymerase chain reaction (PCR) and sequencing analyses (see Methods).

### Infection intensity

The intensity of infection was quantified as the number of oocysts per gram (OPG) of faeces according to the previously published method of Kváč et al. [19]. In brief, the slide was weighed to the nearest 0.001 g before and after the faecal smear to determine the mass of faecal material. After ACMV staining, all oocysts present in the smear were counted microscopically, and the infection intensity was expressed as the number of OPG of faecal material. The resulting OPG value was calculated as the average of three smears of the same faecal sample.

### Oocyst morphometry

Oocysts of *Cryptosporidium* sp. lizard genotype (isolates liz_CZK_1.0 and liz_CZK_2.0) obtained from naturally infected geckos and isolates liz_CZK_2.1–2.3 obtained from experimentally infected hosts using liz_CZK_2.0 as inoculum were purified by sucrose and caesium chloride gradient centrifugation [20] for subsequent morphological analysis. For each isolate, the length and width of 100 oocysts were measured and a shape index calculated. As a reference, oocysts of *Cryptosporidium parvum* isolate HA (*n* = 100), originally isolated from a naturally infected calf and maintained for two decades in SCID [severe combined immunodeficiency] mice at the Biology Centre of the Czech Academy of Sciences (BC CAS), Czech Republic, were included. All measurements were performed by the same researcher using differential interference contrast (DIC) microscopy at 1000× magnification (Olympus BX51, Olympus Corporation, Tokyo, Japan). Photomicrographs for morphometric analysis were analysed using Olympus CellSens Entry 2.1 software in combination with an Olympus Digital DP73 colour camera (Olympus Corporation). Additional staining and labelling techniques were used to visualise the oocysts, including ACMV, modified Ziehl–Neelsen (ZN) [21], auramine phenol (AP) [22] and Cy3-labelled mouse monoclonal antibodies targeting antigenic sites of the oocyst wall (A400Cy2R-20X, Crypt-a-Glo, Waterborne, Inc., New Orleans, LA, USA).

### Molecular characterisation and sequencing

 Total genomic DNA (gDNA) was isolated from 5000 purified oocysts or 200 mg of faecal material using the GeneAll^®^ Exgene™ Stool DNA Mini Kit (GeneAll Biotechnology Co., Ltd., Seoul, South Korea) and from 100 mg of tissue using the DNeasy Blood & Tissue Kit (QIAGEN, Hilden, Germany), following the manufacturers’ protocols with slight modification. Specifically, sterile 0.5 mm glass beads were added to the microtubes up to the 100 µl mark, followed by the addition of 10 sterile zirconia beads (2 mm in diameter; Next Advance, Troy, NY, USA) to each tube containing the sample and lysis buffer. The samples were then homogenised for 60 s at 5.5 m/s using a FastPrep^®^-24 device (MP Biomedicals, Santa Ana, CA, USA) [23].

The extracted gDNA was stored at −80 °C until further processing. Partial sequences of the genes for the small subunit rRNA (*SSU*), actin, the *Cryptosporidium* oocyst wall protein (*COWP*), the 70-kDa heat shock protein (*HSP70*) and the thrombospondin-related adhesive protein of *Cryptosporidium*-1 (*TRAP-C1*) were amplified using previously published nested PCR protocols and primer sets [24–29]. Both primary and secondary PCR reactions were performed in 50 μl reaction volumes. The primary reaction mixture contained 2 μl of gDNA template, 2.5 U DreamTaq Green DNA Polymerase (Thermo Fisher Scientific, Waltham, MA, USA), 1× PCR buffer, 200 nM of each primer, 200 μM of each dNTP, 2 μl of non-acetylated bovine serum albumin (BSA; 10 mg/ml; New England Biolabs, Beverly, MA, USA) and molecular grade water. The magnesium chloride (MgCl_2_) concentration was adjusted to 6 mM for *SSU* and 3 mM for the other target genes (actin, *COWP*, *HSP70*, *TRAP-C1*). The same conditions were used for the secondary reactions, except that 2 μl of the primary PCR product was used as a template and the MgCl_2_ concentration was set to 3 mM for all genes. Molecular water and gDNA from *Cryptosporidium*-negative faecal samples were used as negative controls, while *C. serpentis* DNA was used as a positive control. PCR products were resolved on 2% agarose gels stained with ethidium bromide, purified using the Monarch^®^ DNA Gel Extraction Kit (New England Biolabs, Ipswich, MA, USA) and sequenced in a commercial laboratory (SeqMe, s.r.o., Dobříš, Czech Republic) using secondary primers.

### Phylogeny analysis

Each gene from each isolate was independently sequenced three times. The resulting Sanger chromatograms were processed and edited using ChromasPro 2.1.8 (Technelysium, Pty, Ltd., South Brisbane, Australia). The nucleotide sequences were aligned with each other as well as with reference sequences from GenBank (https://www.ncbi.nlm.nih.gov) using the online alignment tool MAFFT version 7 (http://mafft.cbrc.jp/alignment/software/). Manual trimming of alignments was performed in BioEdit v.7.0.5. Phylogenetic trees were generated using maximum likelihood (ML) and neighbor-joining (NJ) methods in MEGA X, with model selection based on the best-fitting evolutionary parameters (GTR + G model). Node support was evaluated by bootstrap analysis with 1000 replicates. Pairwise genetic distances, expressed as the number of nucleotide substitutions per site, were also estimated in MEGA X. The sequences collected in this study were deposited in GenBank under the following accession numbers: PV535941–PV535945 (*SSU*), PV550915–PV550919 (actin), PV550920–PV550924 (*HSP70*) and PV550925–PV550929 (*COWP*).

### Transmission studies

Oocysts of the isolate liz_CZK_2.0 were used for the experimental infections. Three adult leopard geckos (*E. macularius*), three adult corn snakes (*P. guttatus*), three SCID mice (*Mus musculus*) and three day-old chicks (*Gallus gallus* f. *domestica*) were each orally inoculated with 5000 oocysts. Prior to inoculation, faecal samples from all adult animals were examined daily for 1 month to confirm the absence of *Cryptosporidium* oocysts and *SSU* rRNA-specific DNA. Geckos and snakes were inoculated alongside their regular feeding. After inoculation, faecal samples were collected daily—or at different intervals depending on the faecal behaviour of the reptiles—and tested for the presence of *Cryptosporidium* oocysts and specific *Cryptosporidium* DNA from the third day post-infection (DPI) using ACMV staining and PCR for the *SSU* rRNA gene, respectively. Mice and chicks were observed for 30 DPI, while geckos and snakes were observed for 50 DPI. The infection intensity was then quantified (see above). Animals were housed individually according to species-specific requirements: rodents in ventilated cages (Tecniplast, Buguggiate, Italy), chickens in boxes and reptiles in terrariums. The chicks were provided with external heat sources during the first 5 days, the reptiles during the entire experiment. The gecko terrariums were equipped with ultraviolet A (UVA) and ultraviolet B (UVB) lighting. All animals had ad libitum access to sterilised water. The geckos were fed every other day with *Acheta domesticus* (crickets) and received vitamin and calcium supplements; the snakes received weekly pre-killed laboratory mice, and the mice and chicks were fed standard diets ad libitum. To ensure biosecurity, the animal keepers wore sterile shoe covers, disposable overalls and gloves when entering the experimental room. The wood chip bedding and all disposable protective clothing were removed and incinerated after use.

### Clinical and pathomorphological examinations

Each animal was monitored for frequency of defecation, and consistency of faeces and body weight were recorded weekly. Extensive tissue samples were collected from a leopard gecko experimentally infected with *Cryptosporidium* sp. lizard genotype. Samples were taken from the oesophagus, stomach, duodenum, proximal, central and distal jejunum, ileum, caecum, large intestine, cloaca, liver, spleen, kidney, trachea, lung and heart. Sterile dissection instruments were used for each anatomical site to avoid cross-contamination. The removed tissues were prepared for histological examination, scanning electron microscopy (SEM) and PCR genotyping (see above). Histological sections (5 μm thick) were stained with haematoxylin and eosin (HE) and periodic acid-Schiff (PAS) and examined under 100–400× magnification using an Olympus IX70 microscope [30]. SEM samples were prepared according to a protocol described previously [31] and analysed using a JSM-7401F scanning electron microscope (JEOL).

### Statistical analysis

Statistical differences in oocyst size between *Cryptosporidium* species/isolates were analysed using Hotelling’s multivariate two-sample *t*-test implemented in the ICSNP package (Tools for Multivariate Nonparametrics) [32] in the statistical environment R, version 4.2.2 [33]. This test evaluates the null hypothesis that the two-dimensional mean vectors of the measured variables do not differ between the compared populations.

## Results

### Sequence and phylogenetic analysis

**Fig. 1:**
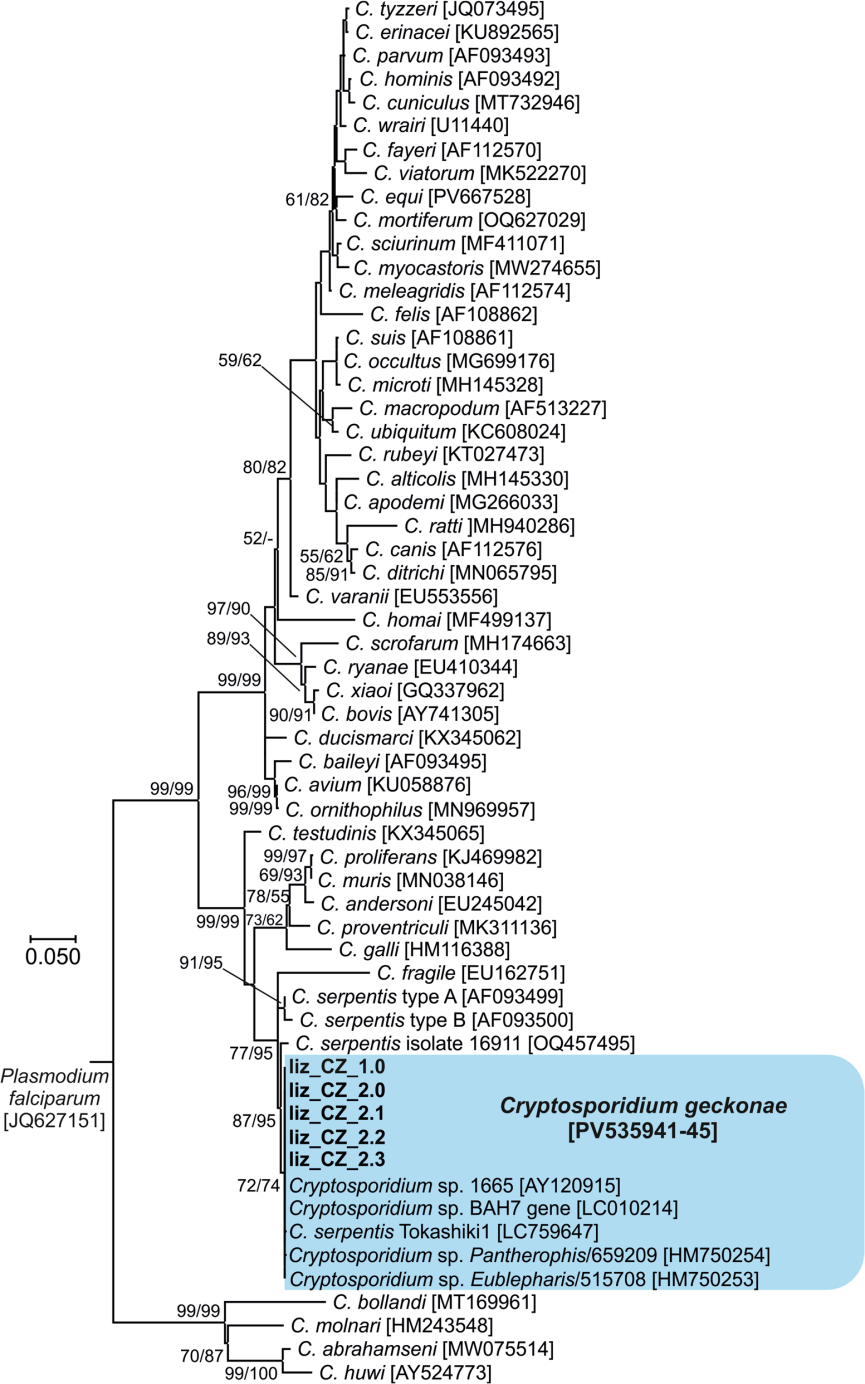
Phylogenetic relationships between the isolates of *Cryptosporidium geckonae* n. sp. (previously known as *Cryptosporidium* sp. lizard genotype) in this study (bold and highlighted) and other *Cryptosporidium* spp. by maximum likelihood (ML)/neighbor-joining (NJ) analyses of the partial small subunit rRNA (*SSU*) gene (GTR + G model). The numbers at the nodes represent the bootstrap values with more than 50% bootstrap support from 1000 pseudoreplicates. The GenBank accession number is in parentheses

**Fig. 2:**
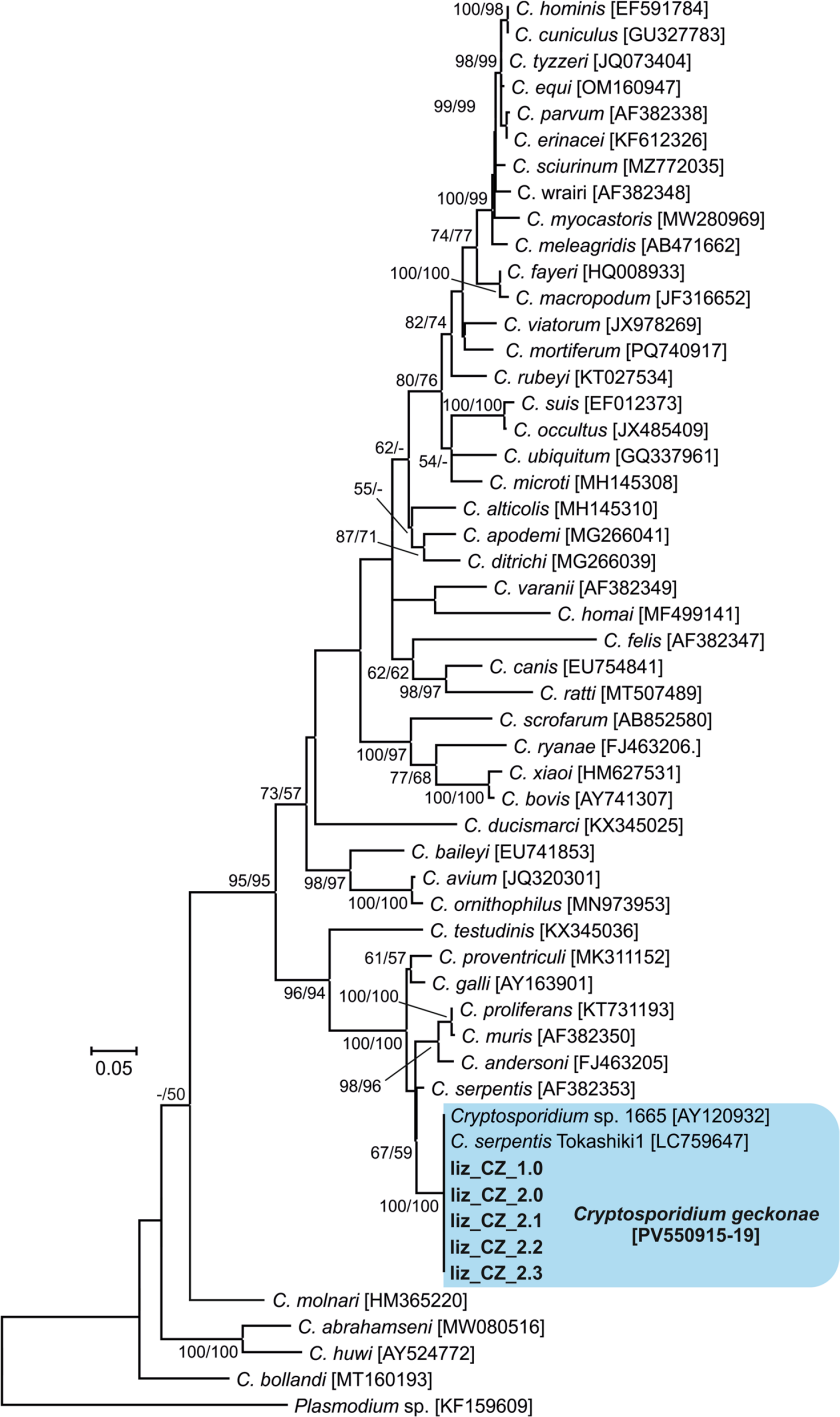
Phylogenetic relationships between the isolates of *Cryptosporidium geckonae* n. sp. (previously known as *Cryptosporidium* sp. lizard genotype) in this study (bold and highlighted) and other *Cryptosporidium* spp. by maximum likelihood (ML)/neighbor-joining (NJ) analyses of the partial actin gene (GTR + G model). The numbers at the nodes represent the bootstrap values with more than 50% bootstrap support from 1000 pseudoreplicates. The GenBank accession number is in parentheses

**Fig. 3:**
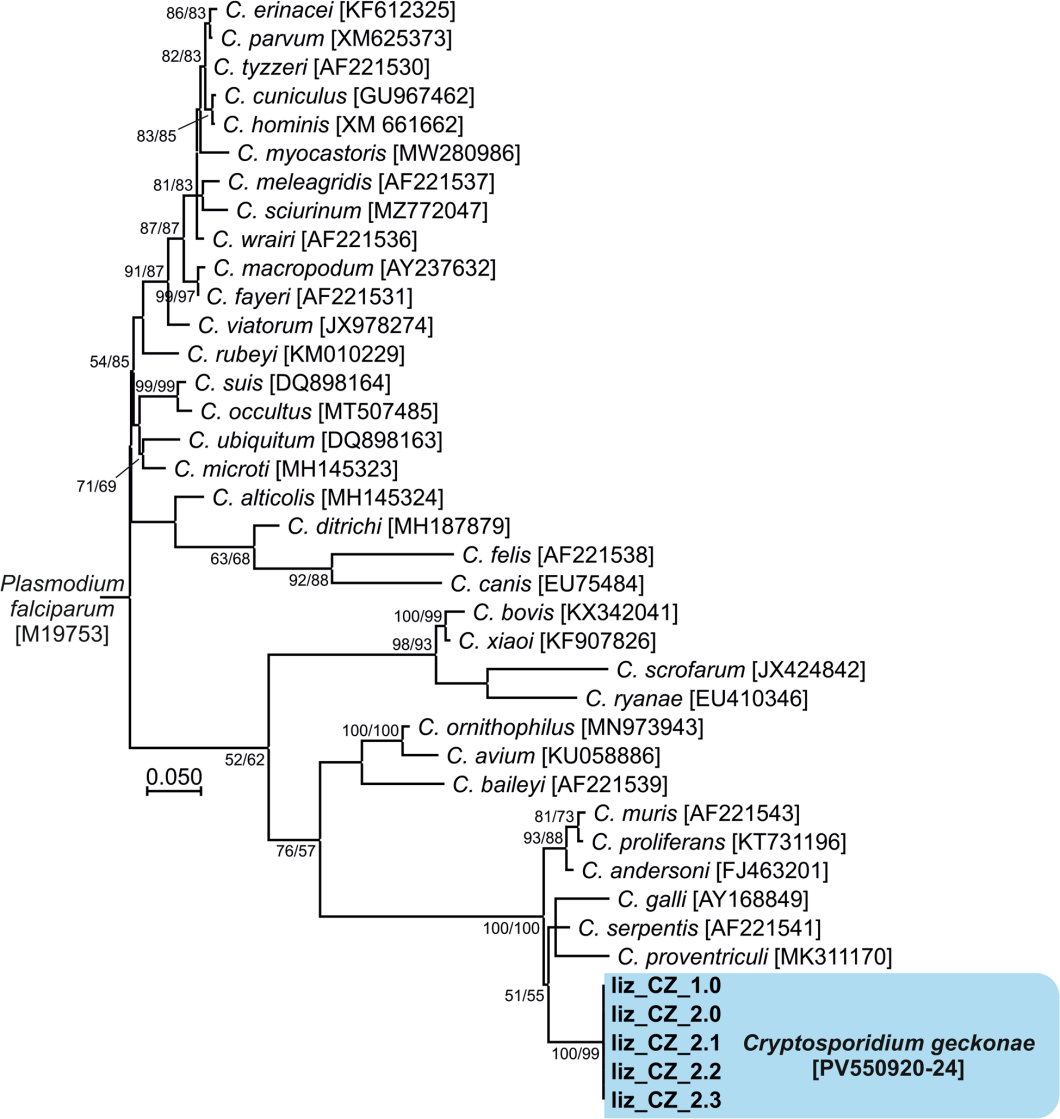
Phylogenetic relationships between the isolates of *Cryptosporidium geckonae* n. sp. (previously known as *Cryptosporidium* sp. lizard genotype) in this study (bold and highlighted) and other *Cryptosporidium* spp. by maximum likelihood (ML)/neighbor-joining (NJ) analyses of the partial 70 kDa heat shock protein (*HSP70*) gene (GTR + G model). The numbers at the nodes represent the bootstrap values with more than 50% bootstrap support from 1000 pseudoreplicates. The GenBank accession number is in parentheses

**Fig. 4:**
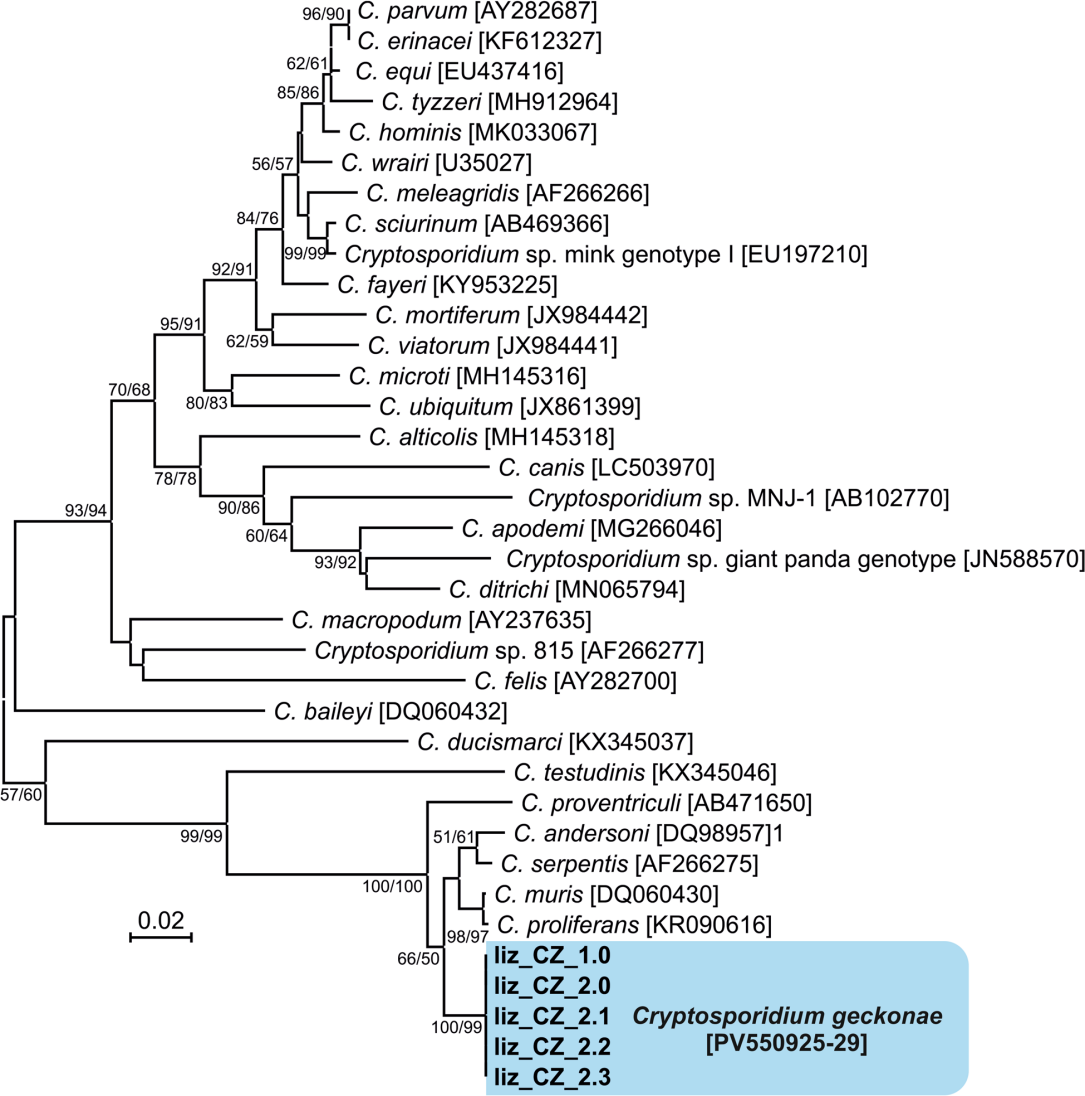
Phylogenetic relationships between the isolates of *Cryptosporidium geckonae* n. sp. (previously known as *Cryptosporidium* sp. lizard genotype) in this study (bold and highlighted) and other *Cryptosporidium* spp. by maximum likelihood (ML)/neighbor-joining (NJ) analyses of the partial *Cryptosporidium* oocyst wall protein (*COWP*) gene (GTR + G model). The numbers at the nodes represent the bootstrap values with more than 50% bootstrap support from 1000 pseudoreplicates. The GenBank accession number is in parentheses

Partial sequences of *Cryptosporidium* sp. lizard genotype genes encoding *SSU* (∼830 bp), actin (∼1050 bp), *HSP70* (∼520 bp) and *COWP* (∼400 bp) were obtained from both naturally infected and experimentally infected animals. Amplification of the genes coding for *TRAP-C1* was unsuccessful. Phylogenetic analyses of the *SSU* and actin genes revealed that isolates liz_CZK_1.0 from the naturally infected smooth knob-tailed gecko and isolate liz_CZK_2.0 from the naturally infected leopard gecko were identical to each other and 100% identical to the *SSU* (AY120915) and actin (AY120932) sequences of the originally described isolate 1665 (Table 1; Fig. 1). All *SSU* and actin sequences obtained from experimentally infected leopard geckos (liz_CZK_2.1–2.3) were identical to each other, to the inoculum used (liz_CZK_2.0) and to isolate 1665 obtained from leopard gecko in the Czech Republic (Table 1; Fig. 1). No sequences of the genes encoding *COWP* and *HSP70* of the *Cryptosporidium* sp. lizard genotype were available in GenBank. Phylogenetic relationships between isolates of *Cryptosporidium* sp. lizard genotype and other *Cryptosporidium* spp. were inferred by ML and NJ analyses at all four loci and revealed similar tree topologies as well as clusters with other previously reported isolates of the *Cryptosporidium* sp. lizard genotype (Figs. 1, 2, 3 and 4).

### Oocyst size and morphology

**Fig. 5:**
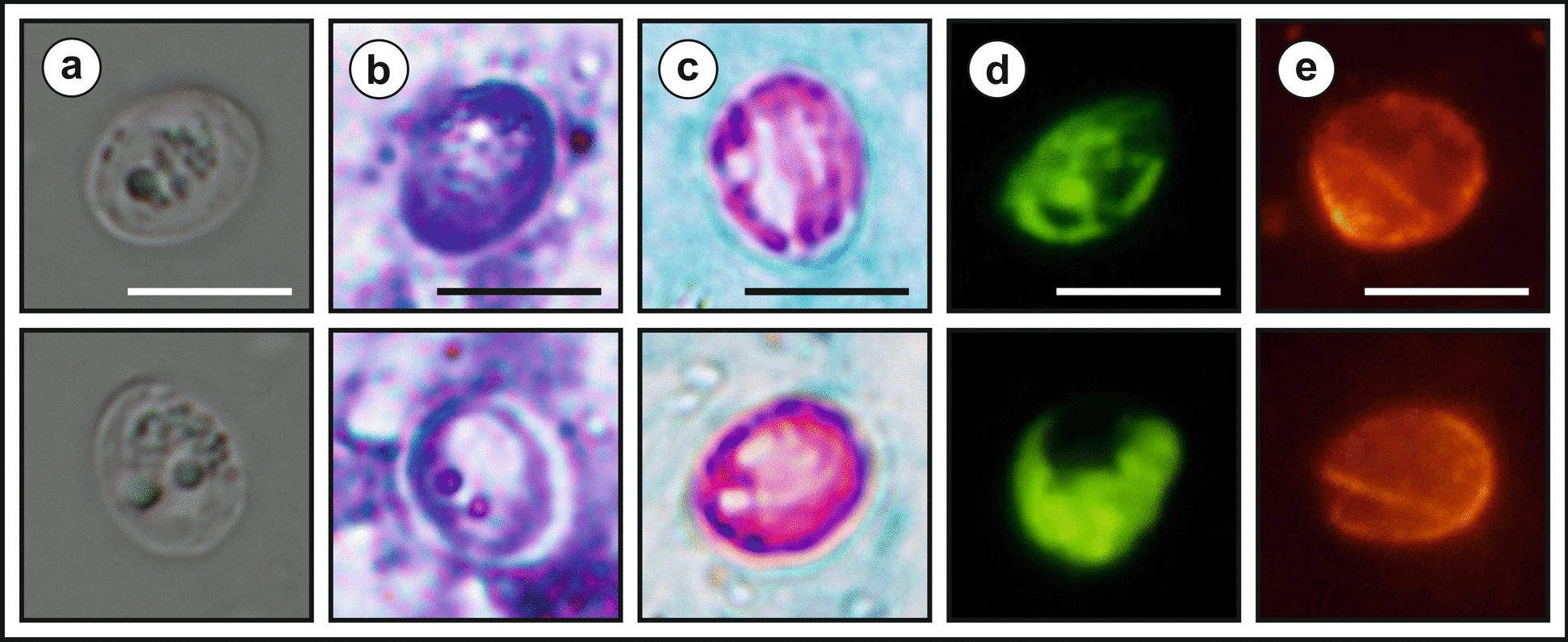
Oocysts of *Cryptosporidium geckonae* n. sp. (previously known as *Cryptosporidium* sp. lizard genotype) **a** in differential interference contrast microscopy, **b** stained by aniline-carbol-methyl violet staining, **c** stained by Ziehl–Neelsen staining, **d** stained by auramine-phenol staining and **e** labelled with anti-*Cryptosporidium* Cy3-conjugated. Bars = 5 μm

The *Cryptosporidium* sp. lizard genotype oocysts of isolates liz_CZK_1.0 and liz_CZK_2.0 obtained from naturally infected geckos measured 5.47–6.20 × 4.32–5.20 μm, with a shape index of 1.07–1.35 (Table 2), and were not statistically significantly different from each other or from isolates obtained from experimentally infected geckos (Table 2). The statistical analysis showed that the isolates did not differ morphometrically from each other (*T*^2^ = 0.1229–1.2103, *df*_1_ = 2, *df*_2_ = 97, *P* = 0.5513–0.9410). Oocysts of *Cryptosporidium* sp. lizard genotype stained with ACVM, ZN and AP staining methods were characteristic of *Cryptosporidium* spp. The intensity of staining with each staining method was similar to that of control staining with *C. parvum* oocysts. Similarly, specific antibodies originally developed against oocyst wall antigens of *C. parvum* interacted with antigens in the wall of oocysts of the *Cryptosporidium* sp. lizard genotype and resulted in a positive fluorescence signal (Fig. 5). The size of the oocysts of the *Cryptosporidium* sp. lizard genotype differs significantly from *C. parvum* (*T*^2^ = 485.8795, *df*_1_ = 2, *df*_2_ = 88.8445, *P* = 1.4573 × 10^–39^).

**Table 2:** Size of *Cryptosporidium geckonae* n. sp. (previously known as *Cryptosporidium* sp. lizard genotype) oocysts recovered from naturally infected smooth knob-tailed gecko (*Nephrurus levis*; liz_CZK_1.0), leopard gecko (*Eublepharis macularius*; liz_CZK_2.0) and leopard geckos (isolates liz_CZK_2.1–2.3) experimentally infected with isolates liz_CZK_2.0

Isolate no.	Length (μm), range (mean ± SD)	Width (μm), range (mean ± SD)	Length/width ratio, range (mean ± SD)
liz_CZ_1.0	5.51–6.19 (5.81 ± 0.18)	4.35–5.19 (4.70 ± 0.19)	1.09–1.35 (1.24 ± 0.06)
liz_CZ_2.0	5.47–6.20 (5.86 ± 0.21)	4.32–5.20 (4.79 ± 0.27)	1.07–1.28 (1.22 ± 0.09)
liz_CZ_2.1	5.51–6.20 (5.84 ± 0.22)	4.37–5.19 (4.76 ± 0.26)	1.11–1.39 (1.23 ± 0.07)
liz_CZ_2.2	5.52–6.18 (5.81 ± 0.19)	4.33–5.14 (4.69 ± 0.23)	1.11–1.38 (1.24 ± 0.08)
liz_CZ_2.3	5.54–6.17 (5.85 ± 0.19)	4.35–5.22 (4.78 ± 0.25)	1.08–1.39 (1.23 ± 0.08)

Length and width of 100 oocysts from each isolate were measured under differential interference contrast at 1000× magnification, and these measurements were used to calculate the length-to-width ratio of each oocyst. SD: standard deviation

### Host specificity and course of infection

**Fig. 6:**
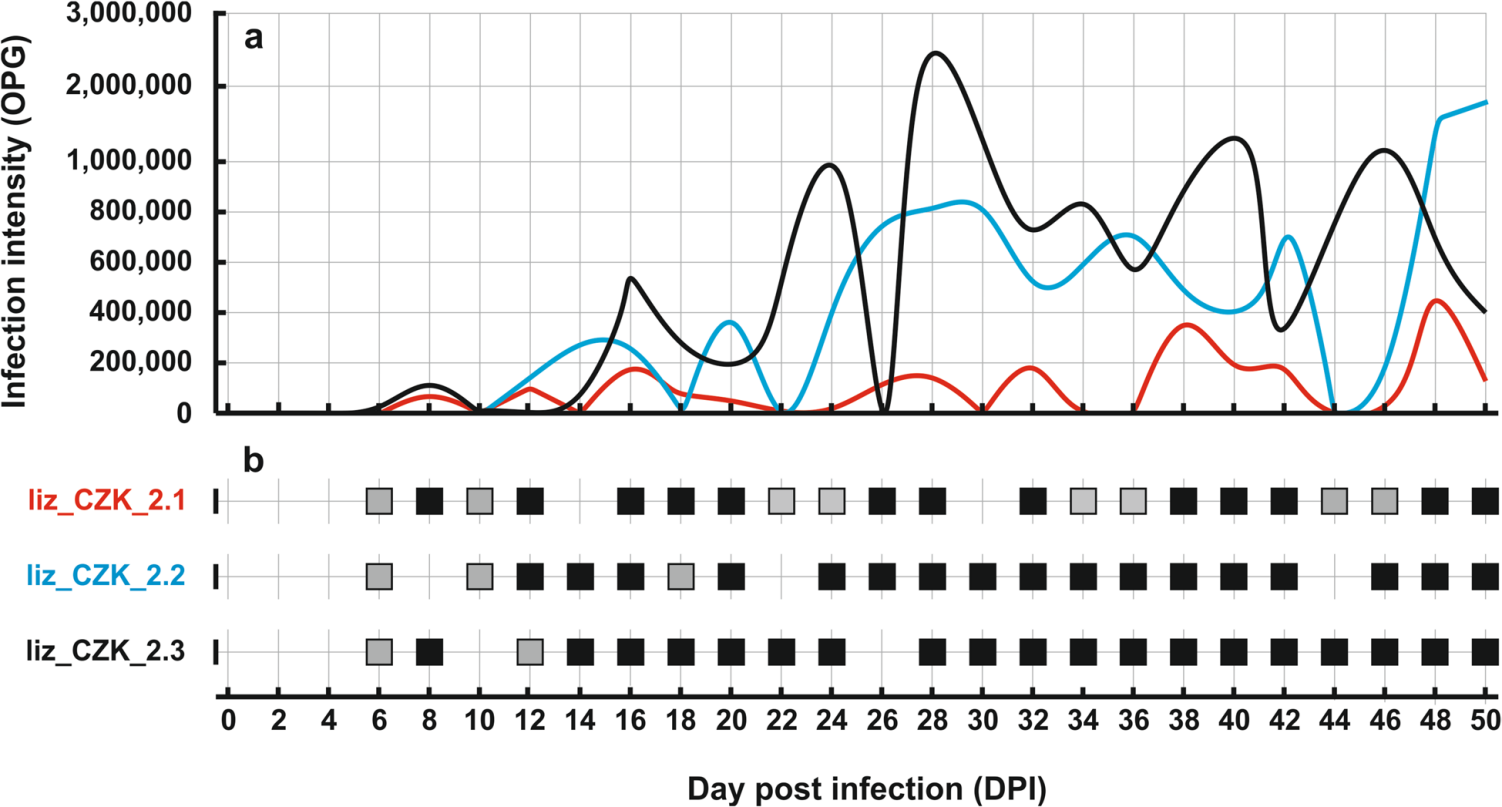
Surveillance of *Cryptosporidium geckonae* n. sp. (previously known as *Cryptosporidium* sp. lizard genotype) infection in leopard geckos (*Eublepharis macularius*) experimentally infected with isolate liz_CZK_2.0 (liz_CZK_2.1–2.3). **a** The severity of infection, determined by quantification of oocyst excretion expressed as oocysts per gram of faeces (OPG). **b** The presence of specific DNA and oocysts, examined by molecular and microscopic methods. Black squares represent samples in which both oocysts and genotype-specific *Cryptosporidium* DNA were detected, while grey squares indicate samples in which only DNA was detected and no oocysts were visible

In mice, chickens and snakes inoculated with 5000 oocysts of the *Cryptosporidium* sp. lizard genotype, no infection was detectable either microscopically or by molecular biology (data not shown). Naturally infected smooth knob-tailed gecko and leopard gecko showed no clinical signs of cryptosporidiosis, and their weight was consistent with species, sex, age and feeding. The owners of these animals reported no health problems. Oocysts of *Cryptosporidium* sp. lizard genotype were infectious to all three inoculated leopard geckos. During the 50-day follow-up period, no clinical signs of infection were observed in these experimentally infected animals. The initial weight of the animals was between 25 and 28 g, and after 50 days they weighed 35–37 g. The course of infection in naturally and experimentally infected geckos is shown in Fig. 6. The prepatent period of *Cryptosporidium* sp. lizard genotype was 6 DPI. Both naturally infected and experimentally infected geckos shed oocysts intermittently. The microscopically detectable infection ranged from 4000 to 2460000 OPG. During long-term observation of naturally infected and experimentally infected geckos, oocysts were shed for more than 200 days (data not shown).

### Localisation of infection and pathological changes

**Fig. 7:**
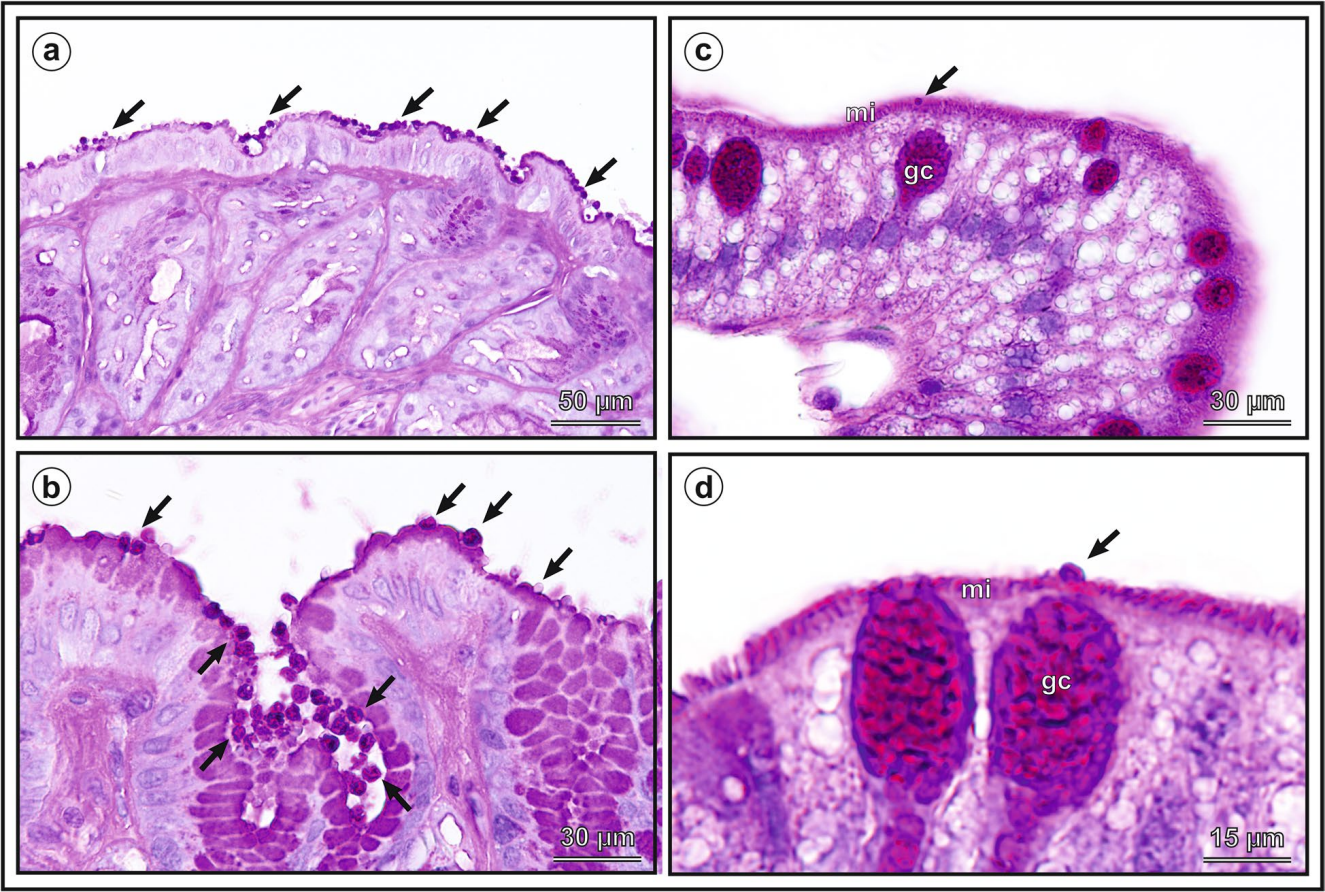
Histological section through the gastric mucosa, stained with Periodic acid-Shiff (PAS). Presence of developmental stages of the *Cryptosporidium geckonae* n. sp. (previously known as *Cryptosporidium* sp. lizard genotype) in the gastric mucosa (**a, b**) and small intestine (**c, d**) of a experimentally infected leopard gecko (*Eublepharis macularius*). **a** massive infection and numerous developmental stages covering the surface of the gastric mucosa (arrows), *b* stomach surface covered with developmental stages (arrow), **c** developmental stage of *C. geckonae* (arrows) embedded in the microvillar layer (mi) of the duodenum near the goblet cell (gc) (arrows), **d** developmental stage of *C. geckonae* (arrows), embedded in the microvillar layer (mi) of the jejunum near the goblet cell (gc) (arrows). Scale bar included in each figure

**Fig. 8:**
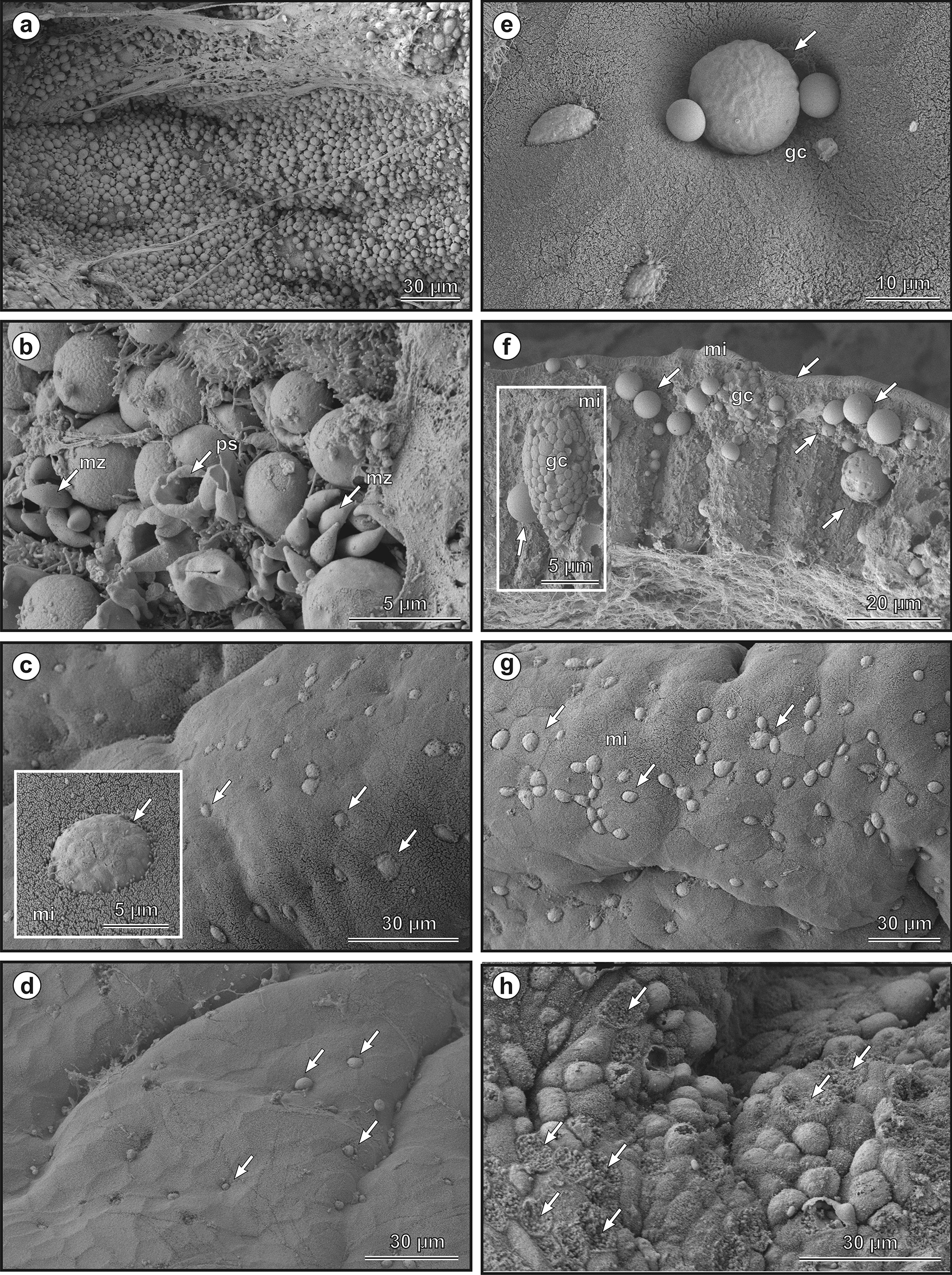
Scanning electron micrographs showing the developmental stages of *Cryptosporidium geckonae* n. sp. (previously known as *Cryptosporidium* sp. lizard genotype) in the stomach (**a, b**), duodenum (**c**), jejunum (**d, e, f**), ileum (**g**) and large intestine (**h**) of leopard gecko (*Eublepharis macularius*). **a** Massive infection and numerous developmental stages covering the surface of the gastric mucosa, **b** surface of the stomach with developmental stages, merozoites (arrows—mz), empty parasitophorous sac after the release of merozoites (arrow—ps), **c** view of cryptosporidia developmental stages (arrows) embedded in the microvillar layer (mi) of the duodenum, **d** surface of the jejunal epithelium with cryptosporidia developmental stages (arrows), **e** developmental stage (arrow) near the goblet cells (gc) in the jejunum, **f** fracture of the jejunum mucosa, with arrows showing developmental stages of cryptosporidia in the microvillar layer (mi) and in deeper parts of the epithelium, where the goblet cell (gc) can be seen in detail, **g** the occurrence of developmental stages in the ileum and embedded in the microvillar layer (mi), **h** the surface of the large intestine, with arrows showing the sites where developmental stages are attached to the epithelium via their feeder organelles. Scale bar included in each figure

Molecular analyses showed the presence of specific DNA of the *Cryptosporidium* sp. lizard genotype in the stomach, small and large intestine, and lung. Subsequent histological and SEM examinations showed the presence of developmental stages in all parts of the digestive tract in which specific DNA was detected (Figs. 7 and 8). No developmental stages were detected in the lungs. Both histological examination and electron microscopy showed that infections with higher intensity were localised in the caudal part of the stomach, closer to the pylorus. In the other parts of the gastrointestinal tract, the intensity of the infection was lower (Figs. 7 and 8). Histological examination showed only isolated stages of development compared to electron microscopy. This difference is due to the area examined with both methods, in favour of electron microscopy. Slight infiltration by mononuclear cells was observed in places in the mucosa. On some slides, the epithelial cells showed changes corresponding to vacuolar degeneration. No significant change in mucus production was observed either in the mucus-forming cells of the gastric mucosa or in the goblet cells of the intestinal mucosa. Likewise, no changes were found in the number or morphology of the goblet cells. Slight oedematous changes were observed in the lamina propria. Also, no elongation of the microvillar layer was observed, as is typical for mammalian hosts/mammalian *Cryptosporidium*, and no epithelial hyperplasia was observed, which is a characteristic histological change in gastric infections with *C. serpentis* in snakes.

### Taxonomic summary

**Family Cryptosporidiidae Léger, 1911**

**Genus** ***Cryptosporidium*** **Tyzzer, 1907**

***Cryptosporidium geckonae*** **n. sp.**

**Syn:** *Cryptosporidium* sp. lizard genotype [10], *Cryptosporidium* sp. 1665 [17], *Cryptosporidium* sp. *Pantherophis*/659209 [11], *Cryptosporidium* sp*. Eublepharis*/515708 [11], *Cryptosporidium* sp. BAH7 [34], *Cryptosporidium serpentis* Tokashiki1 [35].

**Type host:** leopard gecko (*Eublepharis macularius*).

**Other hosts:** smooth knob-tailed gecko (*Nephrurus levis*) (this study), corn snake (*Pantherophis guttatus*) [11], common frog eyed gecko (*Teratoscincus scincus*) [34], Kuroiwa’s ground gecko (*Goniurosaurus kuroiwae sengokui*) [35].

**Type locality:** Písek, Czech Republic.

**Type material:** Faecal smear slides with oocysts stained by ACMV and ZN staining (nos. ACMV1-5/34351 and ZN1-5/34351); SEM specimens of infected stomach (nos. SEM655/2023, SEM656/2023 and SEM567/2020), small intestine (no. SEM658/2023, SEM659/2023, SEM660/2023, SEM661/2023, SEM662/2023, SEM663/2023 and SEM664/2023) and large intestine (nos. SEM665/2023 and SEM666/2023); histological sections of infected stomach (nos. H1/2023, H2/2023 and H3/2023), small intestine (nos. H4/2023, H5/2023, H6/2023, H7/2023, H8/2023, H9/2023 and H10/2023) and large intestine (nos. H11//2023 and H12/2023); gDNA isolated from faecal samples of naturally infected smooth knob-tailed gecko (isolate—liz_CZK_1.0/58816), leopard gecko (liz_CZK_2.0/58976) and experimentally infected leopard geckos (isolate—liz_CZK_2.1/58973; liz_CZK_2.2/60078 and liz_CZK_2.3/60085). All specimens are deposited at the Institute of Parasitology, BC CAS, Czech Republic.

**Site of infection:** stomach, small intestine, large intestine, cloaca, lung (present study).

** Prepatent period:** 6 DPI (this study).

**Patent period:** more than 200 days.

**Representative DNA sequences:** Representative nucleotide sequences of *SSU* [PV535941], actin [PV550915], *HSP70* [PV550920] and *COWP* [PV550925] genes are deposited in the GenBank database.

**ZooBank registration:** The Life Science Identifier (LSID) for this publication is urn:lsid:zoobank.org:pub:5C326765-3F52-41 EB-977F-2FC6361EFE69 (link: https://zoobank.org/References/5c326765-3f52-41eb-977f-2fc6361efe69). The LSID for the newly described species *Cryptosporidium geckonae* n. sp. urn:lsid:zoobank.org:act:D9E205AC-C0A7-4BF1-A606-4F067BE6D07B (https://zoobank.org/NomenclaturalActs/D9E205AC-C0A7-4BF1-A606-4F067BE6D07B).

**Etymology:** As this species has been detected almost exclusively in leopard geckos and we have experimentally verified the susceptibility of this host, the species name *geckonae* is formed from the word “gecko” according to the rules of the International Commission on Zoological Nomenclature (ICZN) and Latin grammar.

**Description:** The oocyst wall is smooth and colourless. The residual body is clearly visible under DIC imaging at 1000× magnification. Staining with specific methods revealed small granules and four sporozoites, which can be observed sporadically. A suture is not recognisable. Sporulated oocysts measure 5.47–6.20 (5.86 ± 0.21) × 4.32–5.20 (4.79 ± 0.27) with a length-to-width ratio of 1.07–1.28 (1.22 ± 0.09) (Fig. 5).

**Differential diagnosis:** Oocysts of *C. geckonae* are stained by ACMV and ZN staining methods and labelled with genus-specific antibodies targeting the *Cryptosporidium* oocyst outer wall antigenic sites, similar to other *Cryptosporidium* spp. (Fig. 5). *Cryptosporidium geckonae* can be differentiated genetically from other *Cryptosporidium* spp. based on nucleotide sequences of *SSU*, actin, *HSP70* and *COWP* genes.

## Discussion

In this study, we obtained isolates of *C. geckonae* from naturally infected geckos and investigated its biological and genetic characteristics. Reports of this species occurring in reptiles are limited. Apart from our study, only four other studies describe the occurrence of this *Cryptosporidium* species in geckos and a snake [11, 17, 34, 35]. The limited number of reports is partly due to the scarcity of studies investigating *Cryptosporidium* in geckos, and the absence of molecular genotyping in earlier publications [36–40]. This study has clearly demonstrated that *C. geckonae* is host-specific for geckos. Experimentally, we were able to transfer the infection from the leopard gecko to other individuals of the same species. Other inoculated hosts (mice, chickens, snakes) were not susceptible to the infection. Noteworthy is the description of *C. geckonae* in a corn snake, reported in Austria [11]. In our study, this snake species showed no susceptibility to infection with *C. geckonae*. Several hypotheses could explain the positive finding in the corn snake in Austria. One possibility is environmental contamination; another is that this individual snake was unusually susceptible to infection. It is not uncommon for host-specific *Cryptosporidium* species to be detected sporadically in non-major host hosts (e.g., *C. muris* in cats, *C. parvum* in birds, *C. suis* or *C. ditrichi* in humans), as well as in non-susceptible hosts such as *C. galli* in bears, *C. felis* in cattle or *C. tyzzeri* in pigs [41–46]. The prepatent period of the *C. geckonae* was consistent across all experimentally infected leopard geckos, with specific DNA/oocysts first detected at 6–8 DPI. While direct comparisons with other studies are not possible, this time frame aligns with the typical developmental cycle of *Cryptosporidium* species, which generally ranges from 3 to 11 days in intestinal species [47–51] and 6–21 days in gastric species [29, 52, 53]. The intensity of infection in the geckos studied was variable during the period observed, with excretion ranging from a few thousand to millions of OPG of faeces, interspersed with intermittent periods of no detectable oocyst shedding. A similar pattern of infection was observed in birds infected with *C. avium*, *C. ornithophilus* and *C. proventriculi*, and in rats infected with *C. occultus*, but the differences between minimum and maximum OPG were not as pronounced [31, 49, 52, 54].

The present work shows that *C. geckonae* lacks tissue specificity. In general, individual *Cryptosporidium* species are thought to have narrow tissue specificity with an affinity for the stomach or segments of the small and large intestine [2, 52, 55–57]. Only some *Cryptosporidium* species in birds, such as *C. baileyi* and *C. avium*, have been shown to infect outside the gastrointestinal tract [49, 58, 59]. However, the primary infection with these avian species is exclusively localised in the intestine of the host. *Cryptosporidium geckonae* is the first *Cryptosporidium* species to escape this classification. Although this *Cryptosporidium* belongs phylogenetically to the gastric species and the gecko stomach was massively parasitised, developmental stages were found in all parts of the gut, and we detected specific DNA in the lung.

Despite the massive infection detected by both histological and SEM methods, we did not observe clinical signs of infection in the geckos. Similarly, a massive digestive tract infection without clinical signs has been described in *C. occultus* [31]. In contrast, *C. serpentis*, another gastric species closely related to *C. geckonae*, is highly pathogenic for snakes. *Cryptosporidium serpentis* typically infects the gastric mucosa and causes hyperplasia, chronic gastritis and clinical signs such as regurgitation, anorexia and progressive weight loss. Affected snakes often show a marked deterioration in health, and the infection can be fatal [60–62]. This comparison highlights the significant differences in pathogenicity of the various *Cryptosporidium* species in the stomach and suggests that host susceptibility and parasite–host interactions play a crucial role in the course of the disease. SEM showed no elongation of microvilli in cells infected with developmental stages of *C. geckonae*, a feature observed in previous in vivo and in vitro studies [2, 31, 63–65].

All standard methods of oocyst staining and labelling successfully detected the oocysts of *C. geckonae* and can be used for microscopic diagnosis, similar to other *Cryptosporidium* species [48, 66–69]. The oocysts of *C. geckonae* measured 5.89 × 4.79 μm. Unfortunately, no further data on the oocyst size of this genotype are available in the literature. Other *Cryptosporidium* species detected in gecko faecal samples have included *C. parvum*, *C. hominis*, *C. varanii*, *C. ditrichi*, *C. serpentis* and a new unnamed genotype reported as *C. serpentis* isolate 16991 [12, 17, 35, 70–72]. It is worth noting that *C. serpentis* isolate 16991 detected in a jewelled gecko (*Naultinus gemmeus*) in New Zealand [70] is genetically distinct from *C. serpentis* types A and B and from *C. geckonae* (Fig. 1). We believe that this is a new genotype infecting geckos, but further analyses are needed. A PCR-positive result for *C. hominis*, *C. parvum* and *C. ditrichi* may indicate environmental contamination, and histological studies were not performed/not found positive on intestinal samples to confirm true parasitism by this *Cryptosporidium* species.

Oocysts of *C. geckonae* are larger than oocysts of *C. parvum* (5.16 × 4.80 μm; this study) and *C. varanii* (5.0 × 4.7 μm), and smaller than oocysts of *C. serpentis* (6.1 × 5.3 μm) [9, 73–75]. However, the differences in oocyst size are too small to allow for reliable differential diagnosis among *Cryptosporidium* species using routine microscopy.

Molecular genotyping provides the only reliable differentiation among *Cryptosporidium* species and genotypes in hosts. Phylogenetic analyses performed in this and other studies on genes encoding *SSU*, actin, *HSP70* and *COWP* confirm that *C. geckonae* is genetically distinct from other species of the genus *Cryptosporidium* [11, 17, 34]. *Cryptosporidium geckonae* forms a separate clade within the gastric cryptosporidia at all loci tested and is most closely related to *C. serpentis*. At the *SSU*, actin, *HSP70* and *COWP* loci, the pairwise distances between *C. geckonae* and *C. serpentis* (0.012, 0.040, 0.047 and 0.025, respectively) were similar to those between other phylogenetically closely related species such as *Cryptosporidium bovis* and *Cryptosporidium xiaoi* (0.003, 0.021, 0.023 and ND, respectively), *C. proliferans* and *C. muris* (0.001, 0.006, 0.012 and 0.003, respectively), and *C. hominis* and *C. parvum* (0.007, 0.018, 0.015 and 0.014, respectively).

## Conclusions

The results of this study indicate that the *Cryptosporidium* sp. lizard genotype is biologically and genetically distinct from all previously described species of the genus *Cryptosporidium*. The observed differences support its consideration as a separate species, for which we propose the name *Cryptosporidium geckonae*.

## Author summary

Parasites of the genus *Cryptosporidium* infect the gastrointestinal tract of many vertebrates, including reptiles. Most *Cryptosporidium* species have narrow host and tissue specificity. A common genotype of *Cryptosporidium*, the lizard genotype, which is mainly found in geckos, has not yet been studied in detail. The authors have studied this genotype in detail using genetic and biological methods. They found that the lizard genotype only infects geckos, but not mice, chickens or snakes. The infected geckos showed no signs of disease, but they shed oocysts of the parasite in their faeces for several months. This genotype of *Cryptosporidium* is not tissue-specific. Developmental stages of the parasite were found throughout the digestive tract and in the lungs. Genetic analyses confirmed that it is a different species from all previously known representatives of the genus *Cryptosporidium*. Based on these results, the authors propose a new name: *Cryptosporidium geckonae*. This discovery contributes to a better understanding of reptile parasites and their evolution.

## Data Availability

All type material and datasets on which the conclusions of the manuscript rely are stored at the Institute of Parasitology, Biology Centre, Czech Academy of Sciences, České Budějovice, Czech Republic. Representative nucleotide sequences generated in this study were submitted to the GenBank database.

## Abbreviations

ACMV: Aniline-carbol-methyl violet
AP: Auramine phenol
BSA: Bovine serum albumin
BC CAS: Biology Centre of the Czech Academy of Science, Czech Republic
*COWP*: *Cryptosporidium* oocyst wall protein gene
DIC: Differential interference contrast
DPI: Days post-infection
gDNA: Genomic DNA
HE: Haematoxylin and eosin
*HSP70*: 70-KDa heat hock protein
ICZN: International Commission on Zoological Nomenclature
NJ: Neighbour-joining
ML: Maximum likelihood
OPG: Oocysts per gram
PAS: Periodic acid-Schiff
PCR: Polymerase chain reaction
rRNA: Ribosomal ribonucleic acid
SD: Standard deviation
SEM: Scanning electron microscopy
SCID: Severe combined immunodeficiency
*SSU*: Small subunit of rRNA
*TRAP-C1*: Thrombospondin-related adhesive protein of *Cryptosporidium*-1
ZN: Ziehl–Neelsen

## References

[1] Nader JL, Mathers TC, Ward BJ, Pachebat JA, Swain MT, Robinson G, et al. Evolutionary genomics of anthroponosis in *Cryptosporidium*. Nat Microbiol. 2019;4:826–36. 30833731 10.1038/s41564-019-0377-x

[2] Tůmová L, Ježková J, Prediger J, Holubová N, Sak B, Konečný R, et al. *Cryptosporidium mortiferum* n. sp. (apicomplexa: cryptosporidiidae), the species causing lethal cryptosporidiosis in Eurasian red squirrels (*Sciurus vulgaris*). Parasit Vectors. 2023;16:235. 37454101 10.1186/s13071-023-05844-8 PMC10349434

[3] Holubová N, Sak B, Hlásková L, Květoňová D, Hanzal V, Rajský D, et al. Host specificity and age-dependent resistance to *Cryptosporidium avium* infection in chickens, ducks and pheasants. Exp Parasitol. 2018;191:62–5. 29959916 10.1016/j.exppara.2018.06.007

[4] Cohn IS, Henrickson SE, Striepen B, Hunter CA. Immunity to *Cryptosporidium*: lessons from acquired and primary immunodeficiencies. J Immunol. 2022;209:2261–8. 36469846 10.4049/jimmunol.2200512 PMC9731348

[5] Ryan UM, Feng Y, Fayer R, Xiao L. Taxonomy and molecular epidemiology of *Cryptosporidium* and *Giardia*—a 50 year perspective (1971–2021). Int J Parasitol. 2021;51:1099–119. 34715087 10.1016/j.ijpara.2021.08.007

[6] Stenger BL, Clark ME, Kváč M, Khan E, Giddings CW, Dyer NW, et al. Highly divergent 18S rRNA gene paralogs in a *Cryptosporidium* genotype from eastern chipmunks (*Tamias striatus*). Infect Genet Evol. 2015;32:113–23. 25772204 10.1016/j.meegid.2015.03.003 PMC4417453

[7] Stenger BLS, Horčičková M, Clark ME, Kváč M, Čondlová S, Khan E, et al. *Cryptosporidium* infecting wild cricetid rodents from the subfamilies Arvicolinae and Neotominae. Parasitology. 2017;145:1–9. 28870264 10.1017/S0031182017001524 PMC6994186

[8] Helmy YA, Krucken J, Abdelwhab EM, von Samson-Himmelstjerna G, Hafez HM. Molecular diagnosis and characterization of *Cryptosporidium* spp. in turkeys and chickens in Germany reveals evidence for previously undetected parasite species. PLoS ONE. 2017;12:e0177150. 28575116 10.1371/journal.pone.0177150 PMC5456029

[9] Pavlásek I, Lávisková M, Horák P, Král J, Král B. *Cryptosporidium varanii* n. sp. (Apicomplexa: Cryptosporidiidae) in Emerald monitor (*Varanus prasinus* Schlegal, 1893) in captivity in Prague zoo. Gazella. 1995;22:99–108, Zoo Praha.

[10] Xiao L, Ryan UM, Graczyk TK, Limor J, Li L, Kombert M, et al. Genetic diversity of *Cryptosporidium* spp. in captive reptiles. Appl Environ Microbiol. 2004;70:891–9. 14766569 10.1128/AEM.70.2.891-899.2004 PMC348785

[11] Richter B, Nedorost N, Maderner A, Weissenböck H. Detection of species in feces or gastric contents from snakes and lizards as determined by polymerase chain reaction analysis and partial sequencing of the 18S ribosomal RNA gene. J Vet Diagn Invest. 2011;23:430–5. 21908271 10.1177/1040638711403415

[12] Pedraza-Diaz S, Ortega-Mora LM, Carrion BA, Navarro V, Gomez-Bautista M. Molecular characterisation of *Cryptosporidium* isolates from pet reptiles. Vet Parasitol. 2009;160:204–10. 19101086 10.1016/j.vetpar.2008.11.003

[13] Rinaldi L, Capasso M, Mihalca AD, Cirillo R, Cringoli G, Caccio S. Prevalence and molecular identification of *Cryptosporidium* isolates from pet lizards and snakes in Italy. Parasite. 2012;19:437–40. 23193530 10.1051/parasite/2012194437 PMC3671454

[14] Kik MJ, van Asten AJ, Lenstra JA, Kirpensteijn J. Cloaca prolapse and cystitis in green iguana (*Iguana iguana*) caused by a novel *Cryptosporidium* species. Vet Parasitol. 2011;175:165–7. 21036480 10.1016/j.vetpar.2010.10.002

[15] Levine ND. Some corrections of coccidian (Apicomplexa: Protozoa) nomenclature. J Parasitol. 1980;66:830–4. 7463253

[16] Koehler AV, Scheelings TF, Gasser RB. *Cryptosporidium* cf. *avium* in an inland-bearded dragon (*Pogona vitticeps*)—a case report and review of the literature. Int J Parasitol Parasites Wildl. 2020;13:150–9. 33088709 10.1016/j.ijppaw.2020.09.004 PMC7560628

[17] Xiao L, Sulaiman IM, Ryan UM, Zhou L, Atwill ER, Tischler ML, et al. Host adaptation and host-parasite co-evolution in *Cryptosporidium*: implications for taxonomy and public health. Int J Parasitol. 2002;32:1773–85. 12464424 10.1016/s0020-7519(02)00197-2

[18] Miláček P, Vítovec J. Differential staining of cryptosporidia by aniline-carbol-methyl violet and tartrazine in smears from feces and scrapings of intestinal mucosa. Folia Parasitol. 1985;32:50. 2580763

[19] Kváč M, Ondráčková Z, Květoňová D, Sak B, Vítovec J. Infectivity and pathogenicity of *Cryptosporidium andersoni* to a novel host, southern multimammate mouse (*Mastomys coucha*). Vet Parasitol. 2007;143:229–33. 16997473 10.1016/j.vetpar.2006.08.031

[20] Arrowood MJ, Donaldson K. Improved purification methods for calf-derived *Cryptosporidium parvum* oocysts using discontinuous sucrose and cesium chloride gradients. J Eukaryot Microbiol. 1996;43:89S. 8822880 10.1111/j.1550-7408.1996.tb05015.x

[21] Henriksen SA, Pohlenz JF. Staining of cryptosporidia by a modified Ziehl-Neelsen technique. Acta Vet Scand. 1981;22:594–6. 6178277 10.1186/BF03548684 PMC8300528

[22] Ley DH, Levy MG, Hunter L, Corbett W, Barnes HJ. Cryptosporidia-positive rates of avian necropsy accessions determined by examination of auramine o-stained fecal smears. Avian Dis. 1988;32:108–13. 3382366

[23] Sak B, Kváč M, Hanzlíková D, Cama V. First report of *Enterocytozoon bieneusi* infection on a pig farm in the Czech Republic. Vet Parasitol. 2008;153:220–4. 18342450 10.1016/j.vetpar.2008.01.043

[24] Jiang J, Alderisio KA, Xiao L. Distribution of *Cryptosporidium* genotypes in storm event water samples from three watersheds in New York. Appl Environ Microbiol. 2005;71:4446–54. 16085835 10.1128/AEM.71.8.4446-4454.2005 PMC1183313

[25] Xiao L, Singh A, Limor J, Graczyk TK, Gradus S, Lal A. Molecular characterization of *Cryptosporidium* oocysts in samples of raw surface water and wastewater. Appl Environ Microbiol. 2001;67:1097–101. 11229897 10.1128/AEM.67.3.1097-1101.2001 PMC92700

[26] Sulaiman IM, Lal AA, Xiao LH. Molecular phylogeny and evolutionary relationships of *Cryptosporidium* parasites at the actin locus. J Parasitol. 2002;88:388–94. 12054017 10.1645/0022-3395(2002)088[0388:MPAERO]2.0.CO;2

[27] Chelladurai JJ, Clark ME, Kváč M, Holubová N, Khan E, Stenger BL, et al. *Cryptosporidium galli* and novel *Cryptosporidium* avian genotype VI in North American red-winged blackbirds (*Agelaius phoeniceus*). Parasitol Res. 2016;115:1901–6. 26818945 10.1007/s00436-016-4930-8

[28] Spano F, Putignani L, Guida S, Crisanti A. *Cryptosporidium parvum*: PCR-RFLP analysis of the TRAP-C1 (thrombospondin-related adhesive protein of *Cryptosporidium* -1) gene discriminates between two alleles differentially associated with parasite isolates of animal and human origin. Exp Parasitol. 1998;90:195–8. 9769250 10.1006/expr.1998.4324

[29] Kváč M, Havrdová N, Hlasková L, Daňková T, Kanděra J, Ježková J, et al. *Cryptosporidium proliferans* n. sp. (Apicomplexa: Cryptosporidiidae): molecular and biological evidence of cryptic species within gastric *Cryptosporidium* of mammals. PLoS ONE. 2016;11:e0147090. 26771460 10.1371/journal.pone.0147090 PMC4714919

[30] Kváč M, Vítovec J. Prevalence and pathogenicity of *Cryptosporidium andersoni* in one herd of beef cattle. J Vet Med B. 2003;50:451–7. 10.1046/j.0931-1793.2003.00701.x 14633200

[31] Kváč M, Vlnatá G, Ježková J, Horčičková M, Konečný R, Hlásková L, et al. *Cryptosporidium occultus* sp. n. (apicomplexa: cryptosporidiidae) in rats. Eur J Protistol. 2018;63:96–104. 29506004 10.1016/j.ejop.2018.02.001

[32] Nordhausen K, Sirkia S, Oja H, Tyler D. CSNP: tools for Multivariate Nonparametrics. 2018. R package version 1.1–1. https://CRAN.R-project.org/package=ICSNP.

[33] R Core Team: R. A language and environment for statistical computing. In: *R Foundation for Statistical Computing*. Vienna, Austria; 2019.

[34] Abe N, Matsubara K. Molecular identification of isolates from exotic pet animals in Japan. Vet Parasitol. 2015;209:254–7. 25801359 10.1016/j.vetpar.2015.02.035

[35] Ootawa T, Toda M, Takahashi H, Saruta T, Murakami Y, Iguchi Y, et al. Record of *cryptosporidium serpentis* from *goniurosaurus kuroiwae sengokui* (Reptilia, Squamata, Eublepharidae) in Tokashikijima, Okinawa Prefecture, Japan. J Vet Med Sci. 2023;85:1142–5. 37558494 10.1292/jvms.23-0186 PMC10600540

[36] Terrell SP, Uhl EW, Funk RS. Proliferative enteritis in leopard geckos (*Eublepharis macularius*) associated with *Cryptosporidium* sp. infection. J Zoo Wildl Med. 2003;34:69–75. 12723803 10.1638/1042-7260(2003)34[0069:PEILGE]2.0.CO;2

[37] Deming C, Greiner E, Uhl EW. Prevalence of *Cryptosporidium* infection and characteristics of oocyst shedding in a breeding colony of leopard geckos (*Eublepharis macularius*). J Zoo Wildl Med. 2008;39:600–7. 19110703 10.1638/2006-016.1

[38] Taylor MA, Geach MR, Cooley WA. Clinical and pathological observations on natural infections of cryptosporidiosis and flagellate protozoa in leopard geckos (*Eublepharis macularius*). Vet Rec. 1999;145:695–9. 10638796

[39] Graczyk TK, Cranfield MR, Bostwick EF. Hyperimmune bovine colostrum treatment of moribund *Leopard geckos* (*Eublepharis macularius*) infected with *Cryptosporidium* sp. Vet Res. 1999;30:377–82. 10478419

[40] Takaki Y, Takami Y, Watanabe T, Nakaya T, Murakoshi F. Molecular identification of *Cryptosporidium* isolates from ill exotic pet animals in Japan including a new subtype in *Cryptosporidium fayeri*. Vet Parasitol Reg Stud Reports. 2020;21:100430. 32862916 10.1016/j.vprsr.2020.100430 PMC7324920

[41] Al-Abedi G, Al-Eodawee E, Khalili S, Gharban H. First molecular genotyping of *Cryptosporidium felis* in cattle, Iraq. Arch Razi Inst. 2022;77:2345–52. 37274886 10.22092/ARI.2022.358621.2271 PMC10237573

[42] Beser J, Bujila I, Wittesjo B, Lebbad M. From mice to men: three cases of human infection with *Cryptosporidium ditrichi*. Infect Genet Evol. 2020;78:104120. 31751756 10.1016/j.meegid.2019.104120

[43] Kváč M, Myšková E, Holubová N, Kellnerová K, Kicia M, Rajský D, et al. Occurrence and genetic diversity of *Cryptosporidium* spp. in wild foxes, wolves, jackals, and bears in central Europe. Folia Parasitol. 2021;2021:002. 10.14411/fp.2021.002 33543733

[44] Xiao LH, Bern C, Arrowood M, Sulaiman I, Zhou L, Kawai V, et al. Identification of the *Cryptosporidium* pig genotype in a human patient. J Infect Dis. 2002;185:1846–8. 12085341 10.1086/340841

[45] Graczyk TK, Fayer R, Trout JM, Lewis EJ, Farley CA, Sulaiman I, et al. *Giardia* sp. cysts and infectious *Cryptosporidium parvum* oocysts in the feces of migratory Canada geese (*Branta canadensis*). Appl Environ Microbiol. 1998;64:2736–8. 9647860 10.1128/aem.64.7.2736-2738.1998 PMC106456

[46] Kváč M, Kestřánová M, Květoňová D, Kotková M, Ortega Y, McEvoy J, et al. *Cryptosporidium tyzzeri* and *Cryptosporidium muris* originated from wild West-European house mice (*Mus musculus domesticus*) and East-European house mice (*Mus musculus musculus*) are non-infectious for pigs. Exp Parasitol. 2012;131:107–10. 22465334 10.1016/j.exppara.2012.03.016

[47] Kváč M, Kestřánová M, Pinková M, Květoňová D, Kalinová J, Wagnerová P, et al. *Cryptosporidium scrofarum* n. sp. (Apicomplexa: Cryptosporidiidae) in domestic pigs (*Sus scrofa*). Vet Parasitol. 2013;191:218–27. 23021264 10.1016/j.vetpar.2012.09.005 PMC3525736

[48] Robinson G, Wright S, Elwin K, Hadfield SJ, Katzer F, Bartley PM, et al. Re-description of *Cryptosporidium cuniculus* Inman and Takeuchi, 1979 (Apicomplexa: Cryptosporidiidae): morphology, biology and phylogeny. Int J Parasitol. 2010;40:1539–48. 20600069 10.1016/j.ijpara.2010.05.010

[49] Holubová N, Sak B, Horčičková M, Hlásková L, Květoňová D, Menchaca S, et al. *Cryptosporidium avium* n. sp. (apicomplexa: cryptosporidiidae) in birds. Parasitol Res. 2016;115:2243–51. 26905074 10.1007/s00436-016-4967-8 PMC4864505

[50] Fayer R, Santín M. *Cryptosporidium xiaoi* n. sp. (Apicomplexa: Cryptosporidiidae) in sheep (*Ovis aries*). Vet Parasitol. 2009;164:192–200. 19501967 10.1016/j.vetpar.2009.05.011

[51] Tzipori S, Smith M, Halpin C, Angus KW, Sherwood D, Campbell I. Experimental cryptosporidiosis in calves - clinical manifestations and pathological findings. Vet Rec. 1983;112:116–20. 6220509 10.1136/vr.112.6.116

[52] Holubová N, Zikmundová V, Limpouchová Z, Sak B, Konečný R, Hlásková L, et al. *Cryptosporidium proventriculi* sp. n. (apicomplexa: cryptosporidiidae) in Psittaciformes birds. Eur J Protistol. 2019;69:70–87. 30981203 10.1016/j.ejop.2019.03.001

[53] Kváč M, Sak B, Kvetoňová D, Ditrich O, Hofmannová L, Modrý D, et al. Infectivity, pathogenicity, and genetic characteristics of mammalian gastric *Cryptosporidium* spp. in domestic ruminants. Vet Parasitol. 2008;153:363–7. 18343038 10.1016/j.vetpar.2008.01.033

[54] Holubová N, Tůmová L, Sak B, Hejzlerová A, Konečný R, McEvoy J, et al. Description of *Cryptosporidium ornithophilus* sp. n. (Apicomplexa: Cryptosporidiidae) as a new species and diversity in farmed ostriches. Parasit Vectors. 2020;13:340. 32641157 10.1186/s13071-020-04191-2 PMC7346416

[55] Lindsay DS, Upton SJ, Owens DS, Morgan UM, Mead JR, Blagburn BL. *Cryptosporidium andersoni* n. sp. (Apicomplexa: Cryptosporiidae) from cattle, *Bos taurus*. J Eukaryot Microbiol. 2000;47:91–5. 10651302 10.1111/j.1550-7408.2000.tb00016.x

[56] Ryan UM, Monis P, Enemark HL, Sulaiman I, Samarasinghe B, Read C, et al. *Cryptosporidium suis* n. sp. (apicomplexa : cryptosporidiidae) in pigs (*Sus scrofa*). J Parasitol. 2004;90:769–73. 15357067 10.1645/GE-202R1

[57] Vítovec J, Hamadejová K, Landová L, Kváč M, Květoňová D, Sak B. Prevalence and pathogenicity of *Cryptosporidium suis* in pre- and post-weaned pigs. J Vet Med B. 2006;53:239–43. 10.1111/j.1439-0450.2006.00950.x 16732883

[58] Kopacz Z, Kváč M, Piesiak P, Szydlowicz M, Hendrich AB, Sak B, et al. *Cryptosporidium baileyi* pulmonary infection in immunocompetent woman with benign neoplasm. Emerg Infect Dis. 2020;26:1958–61. 32687044 10.3201/eid2608.201117 PMC7392468

[59] Current WL, Upton SJ, Haynes TB. The life cycle of *Cryptosporidium baileyi* n. sp. (Apicomplexa, Cryptosporidiidae) infecting chickens. J Protozool. 1986;33:289–96. 3735157 10.1111/j.1550-7408.1986.tb05608.x

[60] Xiao L, Fayer R, Ryan U, Upton SJ. *Cryptosporidium* taxonomy: recent advances and implications for public health. Clin Microbiol Rev. 2004;17:72–97. 14726456 10.1128/CMR.17.1.72-97.2004 PMC321466

[61] Ruggiero PC, Zacariotti RL, Bondan EF, Lallo MA. Prevalence of *Cryptosporidium serpentis* in captive snakes. Cienc Rural. 2011;41:1975–8.

[62] Paiva PR, Grego KF, Lima VM, Nakamura AA, da Silva DC, Meireles MV. Clinical, serological, and parasitological analysis of snakes naturally infected with *Cryptosporidium serpentis*. Vet Parasitol. 2013;198:54–61. 24041484 10.1016/j.vetpar.2013.08.016

[63] Ježková J, Limpouchová Z, Prediger J, Holubová N, Sak B, Konečný R, et al. *Cryptosporidium myocastoris* n. sp. (Apicomplexa: Cryptosporidiidae), the species adapted to the nutria (*Myocastor coypus*). Microorganisms. 2021;9:813. 33921541 10.3390/microorganisms9040813 PMC8069493

[64] Umemiya R, Fukuda M, Fujisaki K, Matsui T. Electron microscopic observation of the invasion process of *Cryptosporidium parvum* in severe combined immunodeficiency mice. J Parasitol. 2005;91:1034–9. 16419745 10.1645/GE-508R.1

[65] Borowski H, Thompson RC, Armstrong T, Clode PL. Morphological characterization of *Cryptosporidium parvum* life-cycle stages in an in vitro model system. Parasitology. 2010;137:13–26. 19691870 10.1017/S0031182009990837

[66] Huang J, Chen M, He Y, Chen H, Huang M, Li N, et al. *Cryptosporidium equi* n. sp. (apicomplexa: cryptosporidiidae): biological and genetic characterisations. Int J Parasitol. 2023;53:545–54. 37150475 10.1016/j.ijpara.2023.02.008

[67] Horčičková M, Čondlová S, Holubová N, Sak B, Květoňová D, Hlasková L, et al. Diversity of Cryptosporidium in common voles and description of *Cryptosporidium alticolis* sp. n. and *Cryptosporidium microti* sp. n. (Apicomplexa: Cryptosporidiidae). Parasitology. 2019;146:220–33. 30012231 10.1017/S0031182018001142 PMC6994189

[68] Čondlová S, Horčičková M, Sak B, Květoňová D, Hlásková L, Konečný R, et al. *Cryptosporidium apodemi* sp. n. and *Cryptosporidium ditrichi* sp. n. (Apicomplexa: Cryptosporidiidae) in Apodemus spp. Eur J Protistol. 2018;63:1–12. 29360041 10.1016/j.ejop.2017.12.006

[69] Elwin K, Hadfield SJ, Robinson G, Crouch ND, Chalmers RM. *Cryptosporidium viatorum* n. sp. (apicomplexa: cryptosporidiidae) among travellers returning to Great Britain from the Indian subcontinent, 2007–2011. Int J Parasitol. 2012;42:675–82. 22633952 10.1016/j.ijpara.2012.04.016

[70] Garcia-R JC, Pita AB, Velathanthiri N, Pas A, Hayman DTS. Mammal-related *Cryptosporidium* infections in endemic reptiles of New Zealand. Parasitol Res. 2023;122:1239–44. 36959486 10.1007/s00436-023-07824-4 PMC10097775

[71] Dellarupe A, Unzaga JM, More G, Kienast M, Larsen A, Stiebel C, et al. *Cryptosporidium varanii* infection in leopard geckos (*Eublepharis macularius*) in Argentina. Open Vet J. 2016;6:98–101. 27419102 10.4314/ovj.v6i2.5 PMC4935766

[72] Louro M, Hernandez L, Antunes J, Madeira de Carvalho L, Pereira da Fonseca I, Gomes J. *Cryptosporidium* spp. in reptiles: Detection challenges, molecular characterization and zoonotic risk. Food Waterborne Parasitol. 2025;40:e00272. 10.1016/j.fawpar.2025.e00272 PMC12182340 40546388

[73] Pavlásek I, Ryan U. *Cryptosporidium varanii* takes precedence over *C. saurophilum*. Exp Parasitol. 2008;118:434–7. 17945215 10.1016/j.exppara.2007.09.006

[74] Koudela B, Modrý D. New species of *Cryptosporidium* (Apicomplexa: Cryptosporidiidae) from lizards. Folia Parasitol. 1998;45:93–100.

[75] Upton SJ, McAllister CT, Freed PS, Barnard SM. *Cryptosporidium* spp. in wild and captive reptiles. J Wildl Dis. 1989;25:20–30. 2915400 10.7589/0090-3558-25.1.20

